# Prevalence and clinical significance of the rare HOXB13 X285K variant in a French Caribbean prostate cancer cohort

**DOI:** 10.1186/s12885-025-15155-z

**Published:** 2025-11-04

**Authors:** Johan Rose-Dite-Modestine, Alexis Vallard, Jean-Samuel Loger, Sylvie Merle, Laurianne Noly, Ainara Martin-Martinez, Mickaelle Rose, Mylène Annonay, Norelyakin Kara, Désire Nimubona, Nathalie Leclerc, Catherine Ledoux, Sarah Malsa, Xavier Promeyrat, Georges Comlan, Mélanie Percot, Odile Béra, Eléna Lihiouel, Céline Minchaca, Emeline Colomba, Régine Marlin

**Affiliations:** 1 Urology department, Mangot Vulcin Hospital, CHU Pierre Zobda Quitman, Fort-De-France, Martinique; 2 Radiation Oncology department, Clarac Hospital, CHU Pierre Zobda Quitman, Fort-De-France, Martinique; 3 Molecular Biology department, CHU Pierre Zobda Quitman, Fort-De-France, Martinique; 4 Clinical Research and innovation, CHU Pierre Zobda Quitman, Fort-De-France, Martinique; 5 Martinique Regional Oncology Platform, Fort-De-France, Martinique; 6 Medical Oncology department, CHU Pierre Zobda Quitman, Fort-De-France, Martinique; 7 Oncogenetic department, Clarac Hospital, CHU Pierre Zobda Quitman, Fort-De-France, Martinique

**Keywords:** Aggressive prostate cancer, HOXB13 X285K, African descent, Health disparities

## Abstract

**Background:**

The *HOXB13* gene has been associated with hereditary prostate cancer (PCa), with rare germline variants linked to early-onset and aggressive forms of the disease. While the G84E variant has been well-characterized in Caucasian populations, the clinical relevance of the X285K variant—primarily found in individuals of African ancestry—remains unclear. This study aimed to determine the prevalence of HOXB13 X285K in a French Caribbean cohort and explore its association with adverse clinical features.

**Methods:**

We conducted a prospective cohort study including 465 men diagnosed with prostate cancer. Germline sequencing of *HOXB13* was performed using Sanger methodology to identify the presence of the X285K variant.

**Results:**

The HOXB13 X285K variant was identified in 5 patients (1.07%). All carriers had clinically significant disease. Among them, three presented with *de novo* metastatic prostate cancer, and two with intermediate-risk localized disease experienced early biochemical recurrence (at 22 and 34 months). In contrast, recurrence-free survival exceeded 85% at 50 months in the overall cohort.

**Conclusions:**

Although limited by small sample size, these findings suggest a possible link between the HOXB13 X285K variant and early tumor progression. This highlights the importance of screening for this rare variant, particularly in patients of African descent who are underrepresented in genomic studies, to support improved risk stratification and personalized management.

## Background

Disparities in access to cancer care remain a significant issue. Cancer epidemiology in the French overseas territories (FOT) differs significantly from mainland France [1]. Indigenous populations are underrepresented in clinical trials, and little is known about their profiles and outcomes. Martinique, which is a Caribbean FOT made up mainly of people of African origin, has the highest incidence of prostate cancer (PCa), with approximatively 540 new cases per 380,000 inhabitants [2]. These data reflect the high incidence of PCa generally described among black North Americans and Caribbean people [3, 4]. Additionally, the highest age-standardized mortality rates are found in the Caribbean, parts of South America, and in the western, southern, and central regions of Africa [3, 4]. These data underline the need for targeted research to better understand the genetic, environmental and socio-economic factors contributing to this elevated risk in African descent.

Several factors have been widely described as risk factors in PCa [5]. Age is a well-established risk factor for PCa. Recent data from the Martinique cancer registry show that more than one-third of cases are diagnosed under 65 years of age [2]. Family history has also been described as a factor that can increase the risk of developing PCa and having an advanced form of the disease, with the risk increasing based on the familial degree and the number of affected individuals [6]. In addition, a genetic predisposition has been reported in PCa which is considered one of the most inheritable forms of cancer. Extensive twin studies have estimated a heritability at 58% [7]. This high rate of heritability indicates that genetic factors play a substantial role in the risk of developing PCa. Genetic susceptibility can be categorized into genetic factors that are rare but have high penetrance, and those that are more common but generally exhibit lower penetrance. Several studies have reported that DNA damage response genes contribute to the development of early-onset and/or aggressive PCa [8, 9]. *BRCA2* mutations represent the most frequent event [9] with a prevalence of 5.3% in men with metastatic PCa [9, 10]. Recently, other genes have been described in early and/or aggressive forms of the disease. Among them, *HOXB13* which has been described in familial PCa with the detection of the recurrent G84E mutation in four prostate families [11]. This rare mutation most common among men of Western European descent is associated with a significantly increased risk of hereditary PCa particularly among young men [11, 12]. More recently, we reported the HOXB13 X285K, a rare mutation identified in cohorts of African ancestry within genetic databases [13]. Our study consisted of the analysis of 46 early-onset PCa (< 51 years) which resulted in the identification of three *HOXB13* c.853delT mutations. This variant is a deletion of thymine that occurs within stop codon, leading to a stop loss mutation that lengthens the protein by 96 amino acids, i.e. 1/3 of the protein. The association between the HOXB13 X285K with PCa was subsequently confirmed among African American men [14–16]. HOXB13 is a key lineage homeobox transcription factor that plays an essential role in prostate differentiation [17, 18]. Several studies have suggested that alterations in HOXB13 may be involved in the development and progression of PCa. HOXB13 appears to be a co-regulator of androgen receptors (AR) involved in homeostasis of the AR-dependent signaling pathway [19–22]. Nevertheless, HOXB13 also appears to be involved in metastatic tumor progression via an AR-independent pathway [23]. Given the limited knowledge about the clinical relevance of rare *HOXB13* variants, we conducted a descriptive study to evaluate the prevalence of the X285K mutation in a cohort of PCa cases from the French Caribbean. By identifying and characterizing carriers of this variant, our goal was to gain preliminary insight into its potential association with specific clinical features or disease trajectories. A better understanding of the distribution and behavior of the X285K mutation may help lay the groundwork for future studies and contribute to the development of more personalized approaches to PCa management in underrepresented populations.

## Methods

### Study design and participants

We conducted a prospective cohort study, in which urologists, oncologists, and oncogeneticists recruited prevalent cases of PCa without restrictions on age, family history, or disease stage. Between April 2021 and July 2024, patients were enrolled either at diagnosis or during a follow-up visit, according to their clinical trajectory. The study was approved by the *Committee for the Protection of Individuals Sud-Méditerranée IV* (ID-RCB/EudraCT: 2020-A01336-33). All patients gave their written informed consent before inclusion in the study. They provided familial and clinical information, including family history of prostate, breast, and other cancers, as well as medical history and demographic data. Ancestry was one of the inclusion criteria, thus all subjects were of Martinican descent. To explore variations in clinical features by age, patients were categorized into three groups: <56 years, 56–65 years, and > 65 years. Detailed clinical information relating to the diagnosis and treatment, including Gleason score, tumor stage, and prostate-specific antigen (PSA) level at diagnosis, was available from medical records. PSA levels were grouped into three categories: (i) < 10 ng/mL, (ii) 10–20 ng/mL and (iii) > 20 ng/mL according to *National Comprehensive Cancer Network* (NCCN) Guidelines [24]. Clinical stage at diagnosis was classified into three groups: (i) localized group (T1-T2a), which includes cancers confined to the prostate, of small size, and limited to one-half or less of one side of the prostate; (ii) localized group (T2b-T2c), which includes cancers confined to the prostate but with more significant involvement, including up to the entire prostate, without invasion of surrounding structures; and (iii) locally advanced group (T3-T4), which includes cancers that have extended beyond the prostate capsule (T3) or invaded adjacent structures such as the bladder or rectum (T4) and regional/metastatic group, which includes cancers that have extended beyond the prostate capsule or invaded adjacent structures, but with no evidence of regional lymph node involvement (N0) or distant metastasis (M0). Patients were classified into NCCN risk group as follows: low-risk, clinical stage T1-T2a, biopsy Gleason score of ≤ 6, and PSA level < 10 ng/mL; intermediate-risk, clinical stage T2b-T2c, biopsy Gleason score 7, or PSA level 10–20 ng/mL; high-risk, clinical stage > T3a, biopsy Gleason score 8–10, or PSA level > 20 ng/mL, according to AUA/ASTRO Guideline [24].

### Molecular analysis and statistics analysis

Screening of the *HOXB13* gene was performed on peripheral blood DNA. Genomic DNA was extracted using the Qiamp DNA Mini Kit (Qiagen, Hilden, Germany). The coding region of *HOXB13* was amplified by PCR using gene-specific primers designed with Primer-BLAST (NCBI), and sequenced by Sanger sequencing on the ABI 3500 DNA analyzer (Thermo Fisher Scientific). Sequencing reactions were carried out with the BigDye Terminator v3.1 Cycle Sequencing Kit according to the manufacturer’s instructions, and analyzed with SeqScape software (Thermo Fisher Scientific). Recurrence-free survival (RFS) was estimated using the Kaplan-Meier method. Patient characteristics were summarized as mean ± standard deviation for quantitative variables and as number (percentage) for qualitative variables. Comparisons between groups were conducted using the Chi-square or Fisher’s exact test, as appropriate. Missing data were analyzed for each variable. For all analyses, a p-value < 0.05 was considered statistically significant. Statistical analyses were performed using GraphPad Prism 10.

## Results

In total, 465 patients were included in the study. We identified the HOXB13 X285K germline mutation in 5 patients, corresponding to an incidence of 1.07% among the patients analyzed.

### Patient’s and tumor’s characteristics

**Fig. 1:**
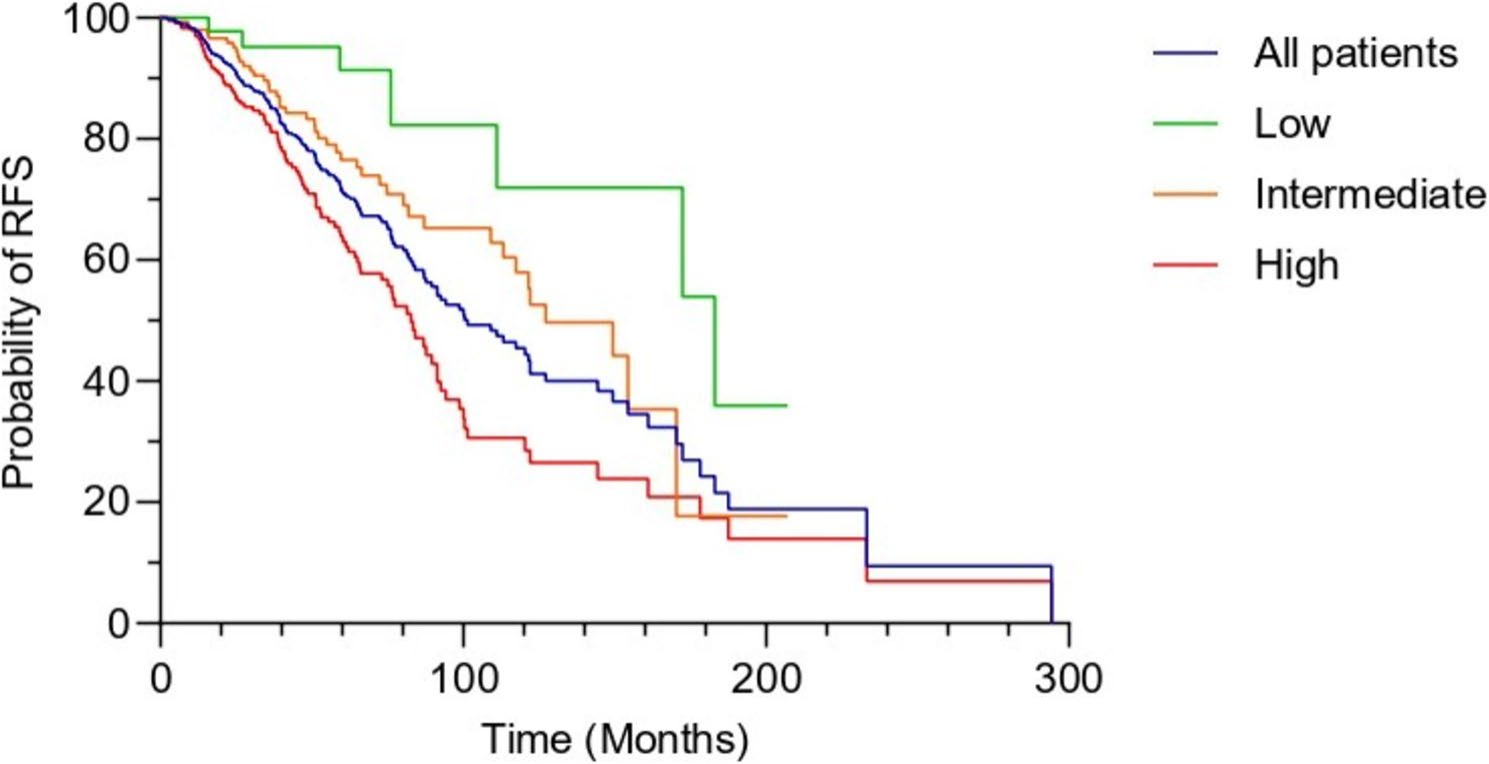
Kaplan-Meier curve for recurrence-free survival according to risk group

Median age at diagnosis was 65 years old (44–92) (Table 1). 15% were diagnosed before the age of 56, and a family history of prostate or breast cancer was reported in 38.2% of cases. Most patients (over 70%) were classified as unfavorable intermediate- to high-risk at diagnosis (Table 1). The 5-year RFS in this population was 76% for intermediate-risk and 63% for high-risk groups (Fig. 1). As expected, disease recurrence was significantly associated with clinical stage (T2b-T2c and > T3a) in localized cases (*p* = 0.00016), while PSA level and Gleason grade were not significantly correlated (Table 2). These baseline characteristics provide a general overview of the cohort from which mutation carriers were identified.

**Table 1:** Clinical and pathological characteristics

Characteristic	Cases *N* (%) 465 (100%)
PSA at diagnosis (ng/ml)	
< 10	210 (45.16)
10–20	103 (22.15)
> 20	135 (29.03)
Unknown	17 (3.65)
Gleason grade	
≤ 6	125 (26.88)
7	218 (46.88)
8–10	113 (24.30)
Unknown	9 (1.93)
Clinical stage	
T1-T2a	159 (34.19)
T2b-T2c	131 (28.17)
>T3a	157 (33.76)
Unknown	18 (3.87)
Risk groups	
Low	51 (10.96)
Intermediate	159 (34.19)
High	244 (52.47)
Unclassified	11 (2.36)
Metatstatic *de novo*	
Yes	85 (18.27)
No	380 (81.73)
Age at diagnosis	
< 56 years	71 (15.26)
56–65 years	171 (36.77)
> 65 years	212 (45.99)
Unknown	11 (2.36)
Family history of prostate and breast cancers	178 (38.19)

**Table 2:** Clinicopathological characteristics of localized PCa associated with relapse

Characteristics	Cases *N* (%) 380 (100%)		
	Relapse 120 (31.57)	No relapse or unknown 260 (68.42)	*p*
PSA at diagnosis (ng/ml)			
< 10	54 (14.21)	144 (37.89)	1.91E-01
10–20	31 (8.15)	61 (16.05)	
> 20	28 (7.36)	44 (11.57)	
Unknown	7 (1.84)	11 (2.89)	
Gleason grade			
≤ 6	33 (8.68)	92 (24.21)	2.50E-01
7	58 (15.26)	123 (32.36)	
8–10	26 (6.84)	38 (10)	
Unknown	3 (0.78)	7 (1.84)	
Clinical stage			
T1-T2a	32 (8.42)	123 (32.36)	1.60E-04
T2b-T2c	45 (11.84)	69 (18.15)	
>T3a	40 (10.52)	51 (13.42)	
Unknown	3 (0.78)	17 (4.47)	
Risk groups			
Low	8 (2.10)	43 (11.31)	7.00E-04
Intermediate	42 (11.05)	116 (30.52)	
High	67 (17.63)	90 (23.68)	
Unclassified	3 (0.78)	11 (2.89)	

### Demographic, genetic characteristics and outcome in *HOXB13 *mutated population

**Fig. 2:**
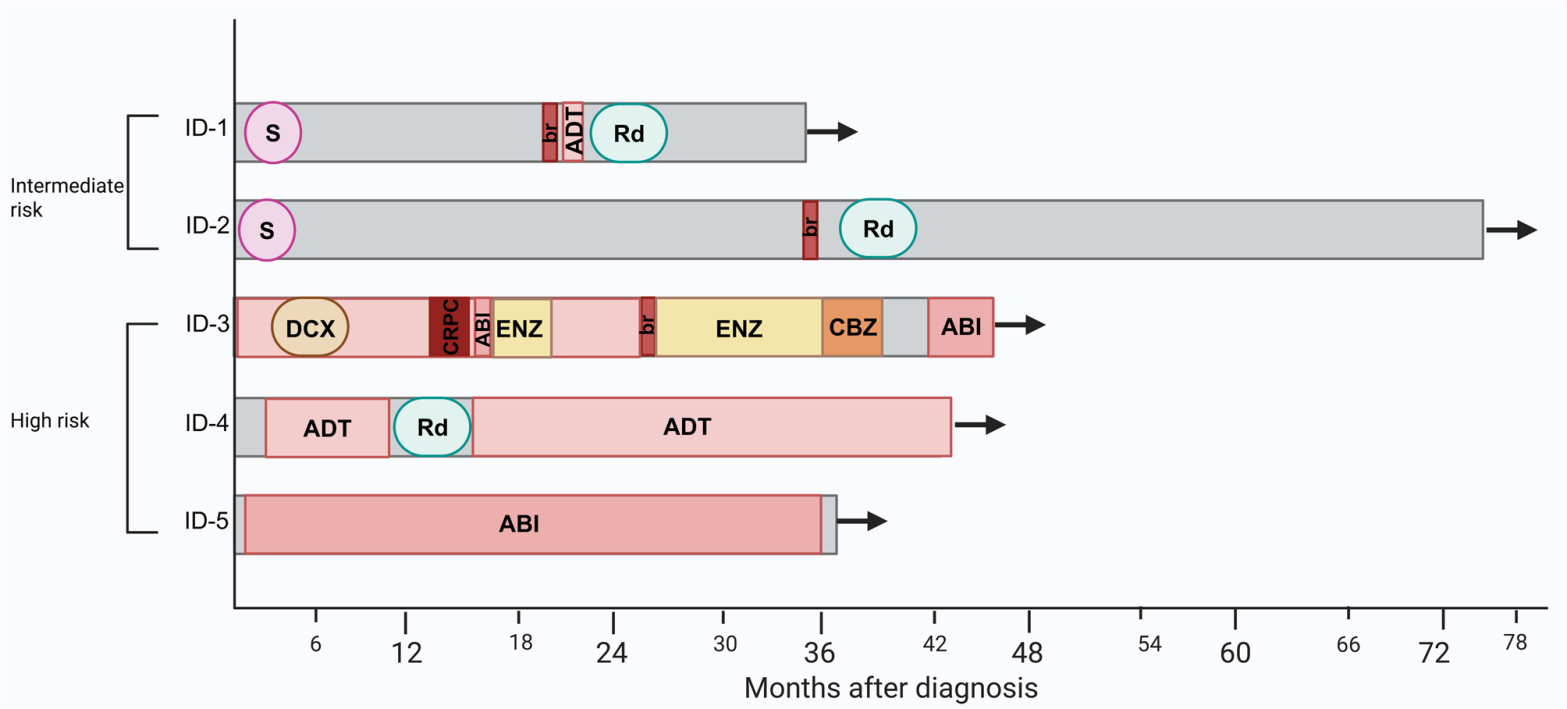
Swimmer plot of patients with HOXB13 X285K. X-axis: months after diagnosis. ABI: Abiraterone. ADT: androgen deprivation therapy. br: biochemical recurrence. CBZ: cabazitaxel. CRPC: castration-resistant prostate cancer. DCX: Docetaxel. ENZ: Enzalutamide. Rd: Radiotherapy. S: Surgery

The pathological and clinical data of patient with the HOXB13 X285K mutation are presented in Table 3; Fig. 2. Two patients were diagnosed at intermediate risk and were treated with radical prostatectomy. Despite initial treatment, they experienced biochemical recurrence at 22 and 34 months post-surgery. Patients diagnosed with advanced-stage PCa (Tumor stage: T3a-T3b, Gleason scores:7–8) presented with *de novo* metastatic disease: one with lymph node metastases and two with lymph node, pulmonary and bone metastases. The patient with lymph node metastases was treated with long-term radiotherapy combined with androgen deprivation therapy (ADT). After a 3-year follow-up, no recurrence has been observed. One of the patients with distant metastases received a combination of ADT, second-generation hormonal therapy, and corticosteroids. He has achieved clinical, radiological, and biochemical remission after 3 years of follow-up. The third patient, also with distant metastases, was initially treated with ADT and chemotherapy. However, he developed castration-resistant disease after 13 months. Subsequent treatments involving hormonal therapy and chemotherapy have been effective in controlling the progression of metastatic disease since the development of castration resistance (Fig. 2). Among the five patients carrying the *HOXB13* X285K variant, one reported a family history of PCa (his father), while no family history of breast or other cancers was observed.

**Table 3:** HOXB13 X285K patient’s characteristics

	Age at diagnosis	PSA Level ng/ml	Gleason grade	TNM Stage	Metastatic site
ID-1	60	12	3 + 3	T2bN0M0	None
ID-2	66	8.6	3 + 3	T2cN0M0	None
ID-3	60	242	4 + 4	T3bN1M1	Lymph node, lung and bone
ID-4	71	7.6	4 + 3	T3aN1M0	Lymph node
ID-5	72	86	4 + 4	T3aN1M1	Lymph node, lung and bone

### Early progression in mutation carriers compared to matched low-risk PCa patients

To better contextualize the clinical behavior of patients carrying the HOXB13 X285K mutation, we identified a subgroup of 40 patients within the cohort who shared similar clinical characteristics to the two mutation carriers diagnosed with intermediate-risk disease: a Gleason score of < 6, a PSA level less than 20 ng/mL, and an initial surgical treatment. Both carriers experienced biochemical recurrence at 22 and 34 months, respectively, prompting a comparative descriptive analysis within this matched subgroup.

**Fig. 3:**
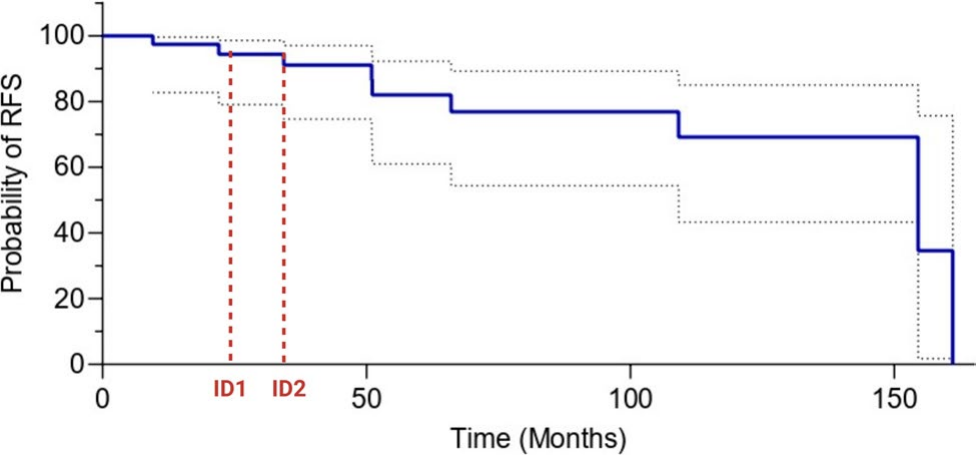
Kaplan–Meier curve of progression-free survival in 40 prostate cancer patients with Gleason score 6, PSA < 20 ng/mL, and surgery initial treatment. The two HOXB13 X285K mutation carriers recurred at 22 and 34 months (indicated by dash lines), whereas the majority of the subgroup maintained progression-free survival beyond 50 months

Kaplan–Meier analysis in this clinically homogeneous subgroup showed a RFS rate of 86.5% at 51 months and 69.2% at 109 months (Fig. 3). The median RFS was not reached at the time of analysis. In contrast, the two mutation carriers progressed earlier than the majority of their clinically matched counterparts. Although statistical testing was not performed due to the small number of mutation carriers, this observation suggests a possible association between the HOXB13 X285K mutation and earlier disease progression in this specific low-risk clinical context.

## Discussion

This study is, to our knowledge, the first prospective investigation to report the prevalence of the HOXB13 X285K mutation in a cohort of PCa patients of African descent. Although the number of mutation carriers is limited, our findings provide valuable descriptive data that may contribute to a better understanding of the potential clinical relevance of this variant. We identified the mutation in 3.5% (3/85) of patients with *de novo* metastatic PCa and in 4.8% (2/42) of intermediate-risk cases who relapsed. Interestingly enough, these latter patients were initially diagnosed with Gleason score 6 tumors and PSA levels below 20 ng/mL—parameters usually associated with a more favorable prognosis. The early recurrence in these cases raises the hypothesis that the X285K may be associated with more aggressive disease behavior or with early tumor escape mechanisms. One of the metastatic cases also presented with a T3aN1 tumor and Gleason score 7. These clinical observations suggest that the mutation could be involved in disease progression even in the absence of high-grade tumor features, emphasizing the need to explore biological mechanisms of progression beyond conventional pathological parameters. These findings are in line with emerging data on HOXB13’s functional roles. The recent work by Nguyen et al. demonstrated that an acetylated form of HOXB-13, conferring a gain-of-function to the protein, enhances the expression of *VEGFA* and *Angiopoietin* genes involved in angiogenesis [25]. Several studies have already implicated HOXB13 overexpression in angiogenesis [26, 27]. The HOXB13 X285K mutation has been described as an activating mutation, the recent in vitro study demonstrated that the X285K mutation leads to activation of the E2F/MYC pathway [16]. While functional studies are needed to demonstrate the impact of the mutation, clinical data of patients support the hypothesis that HOXB13 X285K could contribute to dissemination of tumor cells.

These findings reinforce the importance of investigating hereditary and environmental contributors to disease progression, particularly in populations of African descent, who face a disproportionate burden of aggressive PCa and elevated mortality rates [3, 4]. In this context, the role of environmental exposures such as chlordecone—a persistent organochlorine pesticide widely used in the French Caribbean—deserves particular attention, especially given its known interaction with estrogen receptor alpha (ERα) and its potential implication in angiogenesis-related pathways [28].

Beyond its prognostic implications, the HOXB13 X285K variant could also hold predictive value, particularly in the context of angiogenesis-targeted therapies. While agents such as anti-VEGF antibodies and tyrosine kinase inhibitors have shown promise in several malignancies [29], their efficacy in PCa has been inconsistent, possibly due to the disease’s molecular heterogeneity [30]. Identifying molecular factors that drive tumor escape could help refine patient selection for these therapies. Additionally, since HOXB13 interacts with the AR, this variant may also influence response to hormone-based treatments. In our study, the three patients with *de novo* metastases appeared to respond to AR–targeted therapy (Fig. 2), an observation consistent with the recent report by Kanayama et al., who described favorable responses to such therapies in six *HOXB13* X285K carriers with metastatic hormone-sensitive prostate cancer [31].

Systematic follow-up of mutation carriers and evaluation of their clinical outcomes will be essential to better define the prognostic and predictive value of HOXB13 X285K. Although our study is descriptive and based on a limited number of cases, it contributes to a growing body of evidence supporting the need to explore rare germline variants in diverse populations and to better characterize the mechanisms underlying tumor progression PCa.

Our study has limitations. We did not identify the HOXB13 X285K mutation in early-onset cases. This could be explained by the fact that only 15% of our patients were diagnosed before the age of 56, potentially limiting our ability to detect this mutation in younger cases. Additionally, we present the genotyping of HOXB13 exclusively, as it is the only gene for which we have complete data. However, it is well established that *BRCA2* and other DNA repair genes are involved in certain forms of PCa associated with poor prognosis. Further studies are planned to characterize additional hereditary mechanisms that may be present in our cohort.

## Conclusions

Limited information exists regarding prostate cancer in diverse populations. Our observations suggest a potential involvement of the germline HOXB13 X285Kmutation in the unfavorable progression of PCa within our cohort. We observed a prevalence of 3.5% (3/85) in *de novo* metastatic cases and 4.8% (2/42) in intermediate-risk cases that relapsed, which may indicate clinical relevance, particularly in metastatic disease and early-onset cases. However, due to the small sample size and the descriptive nature of our study, these findings should be interpreted with caution. Further research with larger cohorts is necessary to validate these observations. Nonetheless, these preliminary findings highlight the importance of considering targeted screening strategies in underrepresented populations. Currently, germline *BRCA2* screening is recommended in Caucasian populations, where approximately 5% of cases exhibit this mutation [9]. A similar approach could potentially enhance early risk stratification, prognostic assessment, and personalized therapeutic management in our population. Organizations such as the FDA, EMA, and academic cancer physician groups advocate for increased equity in cancer care access. It is essential that personalized medicine is also accessible to diverse populations. The development of new therapies targeting *HOXB13* mutations could offer significant benefits for patients with advanced PCa harboring this mutation.

## Data Availability

The datasets generated and/or analysed during the current study are available via the link https://bigd.big.ac.cn/gsa-human/browse/HRA013243.

## Abbreviations

ADT: Androgen deprivation therapy
EMA: European Medicines Agency
FDA: Food drug administration
FOT: French overseas territories
NCCN: National Comprehensive Cancer Network
PCa: Prostate cancer
RFS: Recurrence-free survival
